# The Current State of Meniscus Replacements

**DOI:** 10.1007/s12178-024-09902-1

**Published:** 2024-05-14

**Authors:** B. S. van Minnen, T. G. van Tienen

**Affiliations:** 1 Orthopaedic Research Lab, Radboud University Medical Centre, Radboud Institute for Health Sciences, P.O. Box 9101, 6500 HB Nijmegen, The Netherlands; 2 ATRO Medical BV, Liessentstraat 9A, 5405 AH Uden, The Netherlands

**Keywords:** Meniscus replacement, Knee joint meniscus, Meniscus prosthesis, Meniscus implant, Meniscus scaffold

## Abstract

**Purpose of Review:**

The field of meniscus replacement is changing continuously, with new devices emerging and others disappearing from the market. With the current tendency to preserve the knee joint, meniscus implants may become more relevant than ever. The purpose of this review is to provide an overview of the current state of partial and total meniscus replacements that have been developed beyond the academic phase. The available clinical and pre-clinical data is evaluated, and omissions are identified.

**Recent Findings:**

Recent systematic reviews have shown a lack of homogenous clinical data on the CMI and Actifit meniscal scaffolds, especially regarding long-term performance without concomitant surgical interventions. Clinical studies on the medial total meniscus prostheses NUsurface and Artimis are ongoing, with the NUsurface being several years ahead. New techniques for meniscus replacement are rapidly developing, including the Artimis lateral meniscus prosthesis and the MeniscoFix 3D-printed scaffold.

**Summary:**

All evaluated clinical studies point towards improved clinical outcomes after implantation of partial and total meniscus replacements. Long-term data on survival and performance is of low quality for CMI and Actifit and is unavailable yet for NUsurface and Artimis. It is of major importance that future research focuses on optimizing fixation methods and identifying the optimal treatment strategy for each patient group. New techniques for total and partial replacement of the medial and lateral meniscus will be followed with interest.

## Introduction

The medial and lateral meniscus are important structures in the knee joint, with various function including load transmission, shock absorption, stability and joint lubrication [1, 2]. Traumatic and degenerative tears of the meniscus are highly prevalent and often result in pain and other symptoms [3, 4]. Although meniscus repair and preservation are becoming preferred over meniscectomy [5–7], arthroscopic (partial) meniscectomy is still the most commonly performed orthopaedic surgery [8].

As partial or complete loss of the meniscus is associated with knee osteoarthritis and post-meniscectomy pain syndrome (PMPS), treatments are aimed at symptom relief and delay of osteoarthritis [9]. Many patients suffering from PMPS can benefit from nonsurgical treatments, such as braces, physical therapy, medication and viscosupplementation. If nonsurgical management is exhausted, surgical options are limited. High tibial or distal femoral osteotomy may result in positive outcomes but are only feasible in case of malalignment of the knee joint. Knee arthroplasty is generally only considered in older patients with progressed symptomatic osteoarthritis [10, 11].

The most logical solution for a problem that originates in the absence of meniscus tissue, is replacing that meniscus tissue to preserve the joint. Meniscal allograft transplantation (MAT) is one of the more natural replacement strategies. Several different implantation techniques of medial and lateral meniscal allografts have shown to result in improvements in pain, function, stability and activity level and may even decrease the risk of osteoarthritis [12–14]. However, the amount of MAT procedures remains stable and does not significantly grow despite these favorable results [15] and a relatively wide indication [16]. The lack of adoption of MAT may be caused by the technical difficulty of the surgical procedure [17] and the limited availability of (size-matching) allografts in many countries [18, 19].

An off-the-shelf artificial meniscus replacement, in combination with a user-friendly surgical technique, could be a more attractive treatment option for PMPS. Numerous materials including plastics, metals and biomaterials have been used to develop different types of meniscus implants [20–23]. This large variety leads to the question which materials and design concepts are most suitable for this application. In the spectrum of meniscus replacements, the meniscal allograft is probably the most anatomical and biological solution. Meniscal allografts seem to restore native biomechanics and result in positive clinical outcomes despite occurrence of graft shrinkage, graft extrusion and a high re-operation rate [9, 14, 24, 25]. On the other end of the spectrum is the UniSpacer™, a metal self-centering, interpositional spacer without fixation [26]. A recent study showed that 6 out of 8 patients who still had their UniSpacer™ implant were satisfied 10 years after implantation [27]. The high revision rate led to the device being taken off the market in 2011 [23]. One might therefore hypothesize that any interpositional device replacing the meniscus may result in good clinical outcomes, whether it carries all the tibiofemoral joint load and covers the whole tibial plateau or only a very small portion of it. Important prerequisites for positive clinical outcomes are a sufficient long-term survival rate and maintaining knee mobility and stability.

The primary purpose of this review is to provide a detailed overview of the partial and total meniscus replacements that are available on the market or may be in the coming years. While recognizing the progress in the fields of tissue engineering, 3D bioprinting, cell seeding and combinations thereof [28], the focus is on the techniques that have been developed beyond the academic research phase, meaning that clinical studies have been performed or that a company is involved in bringing the device to the market (Table 1).

**Table 1:** Overview of meniscus implants beyond academic research

Device	Manufacturer	Compartment	Clinical data^a^	Approval^b^	Available^c^
CMI	Stryker	Medial & lateral	> 10 years	Europe, US	No
Actifit	Orteq	Medial & lateral	> 10 years	Europe	Yes
MeniscoFix	NovoPedics	Medial	No	No	No
NUsurface	Active Implants	Medial	1 year, ongoing	Europe, Israel	Yes
Artimis (Trammpolin)	ATRO Medical	Medial	< 1 year, ongoing	No	No
		Lateral	Start in 2024	No	No
Total Meniscus Replacement	Orthonika	Medial	No	No	No

^a^Published scientific literature

^b^Regulatory approval for market access

^c^Available for sales on the market

## Meniscal Scaffolds

Meniscal scaffolds are porous structures made from biomimetic materials, intended to stimulate tissue regeneration and to replace (and restore the function of the) missing part of the meniscus [29]. Ingrowth of autologous cells should repair the meniscal fibrocartilage and restore the native biomechanics of the knee joint. The scaffolds are intended to have a degradation rate matched to the rate of meniscal tissue regeneration [30].

Meniscal scaffolds provide an off-the-shelf solution that can be made patient specific by cutting it into the optimal shape for suturing into the meniscal defect [31]. The downside is that these scaffolds are only suitable for patients who underwent partial meniscectomy and have retained a native meniscus peripheral rim and both meniscal horn attachments. Meniscal scaffolds rely on the vascularized portion of the meniscus rim for peripheral support and to facilitate tissue regeneration.

Two meniscal scaffolds have been available on the market: The Collagen Meniscus Implant (CMI; Stryker), which is made of a natural material, and the Actifit® meniscal scaffold (Orteq Sport Medicine), which is made of a synthetic material [29, 32•].

### CMI & Actifit

**Fig. 1:**
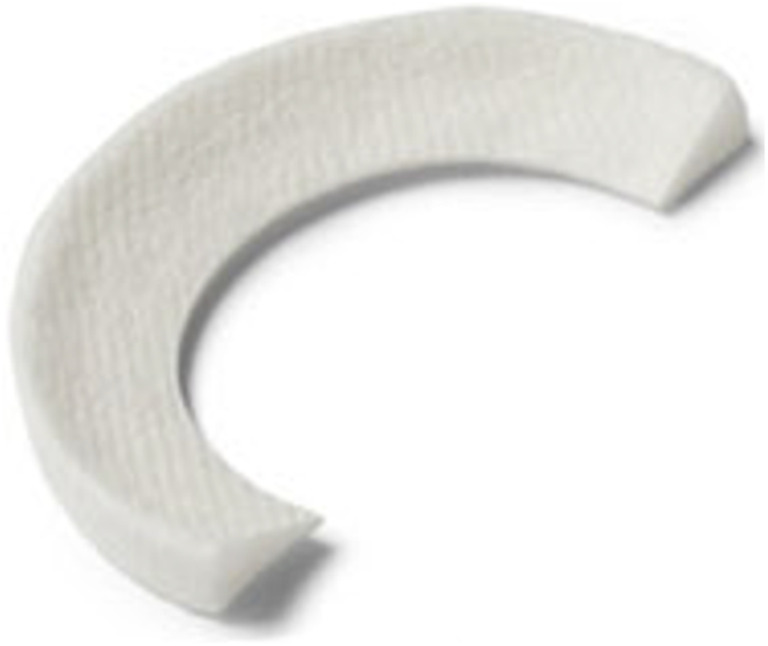
Collagen Meniscus Implant (CMI). Image adapted from Vrancken et al. [20]

**Fig. 2:**
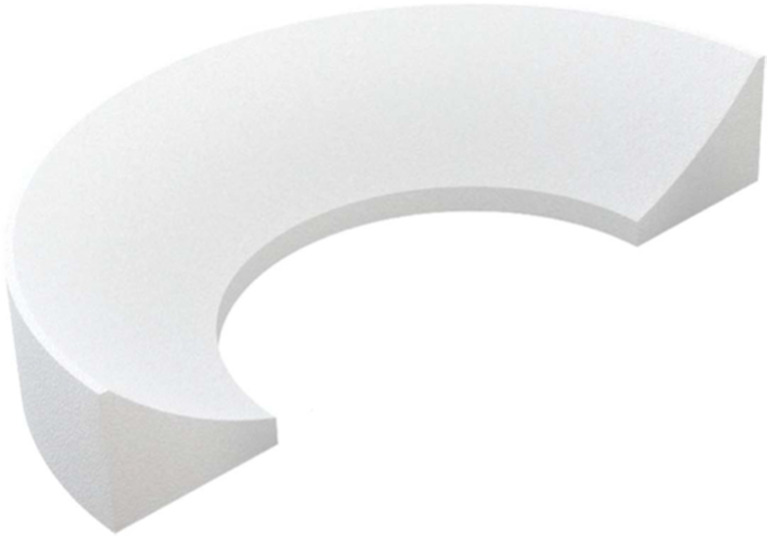
Medial Actifit® Meniscal Scaffold. Image courtesy of Orteq

The CMI (Fig. 1) was developed in the early 1990s and consists of fibrillar type I collagen, derived from bovine Achilles tendons [33, 34]. Development of Actifit (Fig. 2) started shortly after and resulted in a synthetic alternative made of an aliphatic polyurethane [35, 36]. To the authors’ knowledge, both devices are not available in the US and only Actifit is currently available for market sales in the EU.

The most recent systematic reviews reveal that very limited homogenous clinical data is available on meniscal scaffolds, due to lacking standardized outcome measures and a large amount of concomitant surgical procedures [37••, 38•, 39•]. For that reason, benefits of Actifit and CMI for patients suffering from post-meniscectomy pain cannot be demonstrated, especially in the long-term. Moreover, relatively high failure rates of up to 31.8% after five years are reported for the Actifit scaffold [37••, 40].

Although designed to facilitate the natural healing process, recent studies could not demonstrate positive effects of Actifit on chondral degeneration and joint-space narrowing [41–43]. This may be explained by the high frequency of complete resorption of the meniscal substitute, indicating that regeneration of durable meniscus tissue may not occur as intended [44]. For CMI, a large variability in resorption in terms of MRI intensity was reported, together with a high occurrence of chondral defects [45, 46]. Furthermore, MRI analysis of both meniscal scaffolds have shown shrinkage over time [32•].

Despite all of the abovementioned shortcomings almost all individual studies report statistically significant improvements in patient-reported outcome measures (PROMs) on pain and quality of life in the short- and long-term, although one cannot distinguish between the effects of the scaffold and any concomitant procedures [37••, 38•, 39•, 42, 44]. A comparative clinical study including 47 patients found no differences between Actifit and CMI in terms of pain, function and activity level after 10 years of implantation [47].

### MeniscoFix

A novel, collagen-hyaluronan infused 3D-printed polymeric scaffold named MeniscoFix™ has been developed by NovoPedics [48]. Its fiber-reinforced structure allows for attachment to both soft tissue and bone, enabling the possibility for total meniscus replacement and potentially even personalization of the device [49, 50]. So far, biomechanical testing and short-term sheep studies indicate the potential of the MeniscoFix for partial and total meniscus replacement in terms of biomechanics and generation of neomeniscus tissue [48–51]. However, improvements of the fixation method and surgical technique are required to reduce the failure rate in sheep [48]. To the authors’ knowledge, no clinical trial with human subjects has been initiated yet.

## Total Meniscus Replacements

**Fig. 3:**
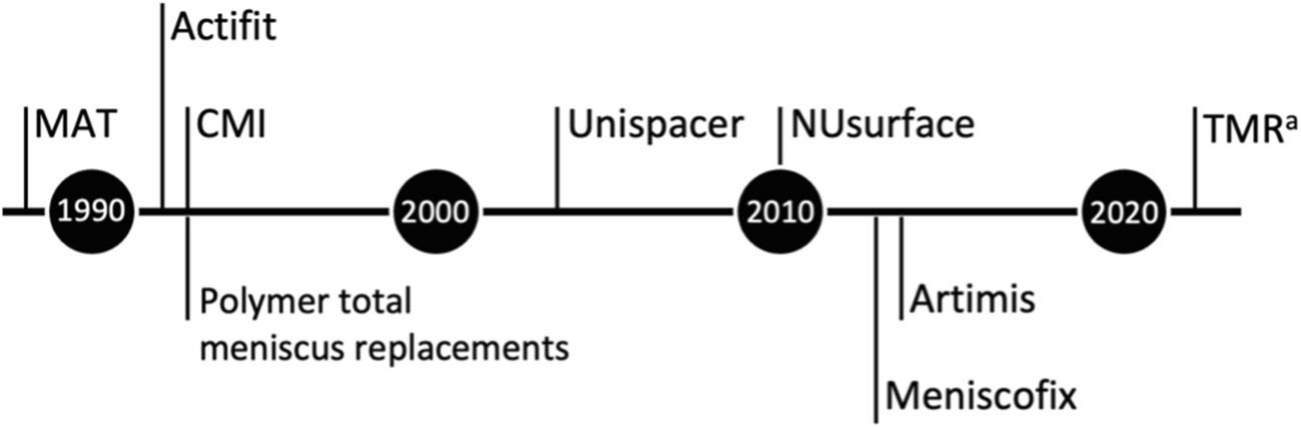
Timeline of first scientific publications on different meniscus replacements [20, 26, 33, 52, 53, 60, 85–87]. ^a^ Total Meniscus Replacement by Orthonika.

Research on permanent, total meniscus replacements made from synthetic polymers has been going on since the 1980s, although the first results were not published until the early 1990s (Fig. 3) [21, 52]. Several materials proved to be insufficient in terms of biocompatibility, mechanical strength or tribological properties. Recently, two permanent, total meniscus replacements have been investigated clinically: NUsurface® (Active Implants) and Artimis® (ATRO Medical). Both implants consist of a relatively new thermoplastic elastomer: Bionate® polycarbonate urethane (PCU; DSM Biomedical).

### NUsurface

**Fig. 4:**
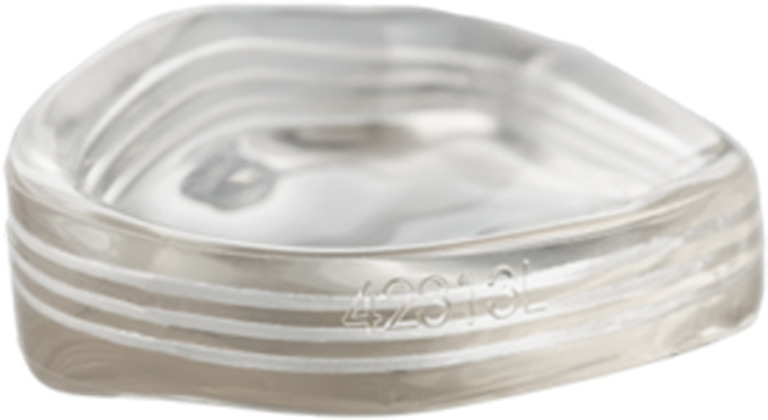
NUsurface® Meniscus Implant. Image courtesy of Active Implants

The NUsurface meniscus implant (Fig. 4) is a free-floating, discoid-shaped implant, intended to replace the medial meniscus [53]. It is made of a flexible PCU, circumferentially reinforced with ultra-high molecular weight polyethylene (UHMWPE) fibers. By applying a wedge-shaped geometry to the complete circumference, including the lateral side, the device becomes self-centering. However, due to the lack of fixation, an intact peripheral meniscus rim of at least 2mm is necessary to prevent dislocation [54]. Additionally, a notchplasty of the lateral side of the medial femoral condyle is sometimes required to avoid impingement on the lateral edge of this medial meniscus replacement [55].

NUsurface has been available on the European market since 2008 [32•], initially with Kevlar fiber reinforcement. After the decision to change to UHMWPE (Dyneema®), two large clinical trials were initiated to validate safety and efficacy: The SUN and VENUS trials. The two studies combined include a total of 242 patients and are referred to as MERCURY [54]. Preliminary results of the MERCURY study group were evaluated by analyzing the outcomes of the first 100 patients with one year follow-up and comparing with a nonsurgical control group [54]. In the implant group, the improvement in KOOS pain compared to baseline (30 points) was significantly larger than in the control group (14 points). It should be noted that, for various reasons, 1-year KOOS data was unavailable for 13% of the included patients. In terms of adverse events, no significant differences were found between the implant group (17%) and the control group (14%).

The latest reported clinical data concerns the complete set one-year outcomes of the VENUS trial [56•], meaning that there is considerable overlap in the two patient populations discussed here. Again, greater improvements in KOOS pain were observed in the implant group than in the nonsurgical control group, with 18% requiring subsequent surgery after receiving the implant. Unfortunately, clinical data with longer follow-up periods has not been published, so long-term survival and effectiveness are yet to be demonstrated.

### Artimis

**Fig. 5:**
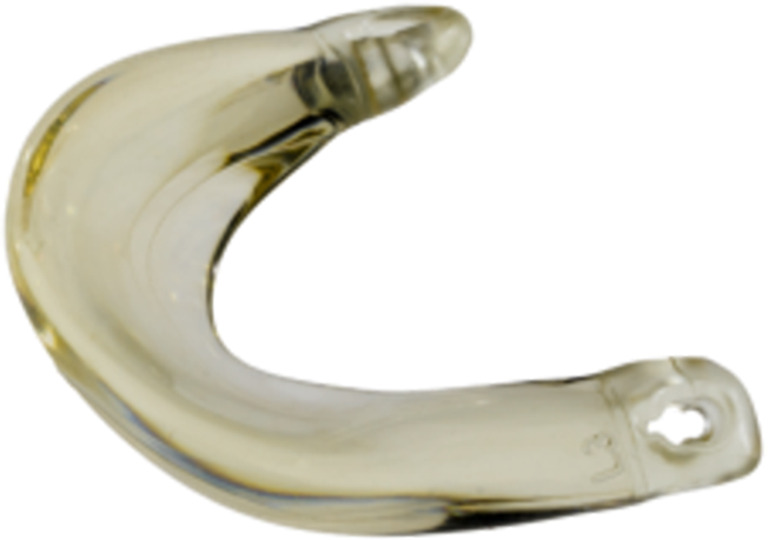
Artimis® Medial Meniscus Prosthesis. Image courtesy of ATRO Medical

The anatomical shape of the Artimis meniscus prosthesis (Fig. 5), previously known as Trammpolin®, is based on the geometry of 35 healthy menisci, derived from MR images [57]. An anterior and posterior horn were added to allow fixation to the tibial plateau [58•]. The cartilage-contacting parts of the first version of the medial meniscus prosthesis were made of a flexible PCU, while the horns and a reinforcement core were made of a stiffer variant of the same PCU. In this first design, attachment to the tibial plateau was achieved by implantation of two titanium fixation screws.

A first-in-human study was initiated with this version of the medial prosthesis, with the intention to include 18 patients [58•]. After five patients, inclusion stopped after the occurrence several device-related adverse events. Three prostheses were removed after implant failure, and one was removed because of persistent pain and a range of motion deficit. The remaining patient started to improve one year after implantation and reported excellent pain scores after two years.

It was hypothesized that the failures and poor clinical outcomes were the result of the rigidity of the meniscus prosthesis and its fixation technique [58•, 59]. A more flexible device was developed, by removing the reinforcement core and replacing the titanium screws with a PET fixation tape. This second-generation prosthesis showed promising pre-clinical results, by significantly reducing tibiofemoral contact pressures after meniscectomy [59]. Last year, a clinical study with 10 patients was initiated and will be expanded with four more patients. One-year data of the first 10 patients is expected soon and will provide valuable insights on the safety and effectiveness of the device. Preliminary results indicate that the prosthesis is able to reduce post-meniscectomy pain and that the meniscus implants stays intact, but also that the fixation technique still needs improvement.

More recently ATRO Medical has developed a lateral total meniscus replacement, also consisting of an anatomically shaped body with reinforced attachment horns and a tape fixation. The first clinical trial to assess the feasibility of the Artimis lateral meniscus prosthesis is expected to start this year, initially including 16 patients with lateral post-meniscectomy pain.

### Total Meniscus Replacement

Orthonika, a spin-out company from Imperial College London, is currently developing a novel, anatomically shaped synthetic total meniscus replacement, reinforced with a fiber matrix [60]. So far, only one pre-clinical study has been published, investigating the biomechanics of a dedicated interference screw fixation system.

## Discussion

Meniscal scaffolds for partial meniscus replacement seem to achieve functional performance despite observations of shrinkage, resorption, and even chondral degeneration [32•, 41–46]. Similarly, meniscal allografts have demonstrated good clinical outcomes despite reports of graft shrinkage and extrusion [9, 14, 24, 25]. It is therefore interesting to determine the mechanism behind the functional performance of meniscus implants and which characteristics eventually lead to pain relief in post-meniscectomy patients.

Even though several meniscus implants obtained market approval many years ago, the quality and consistency of published clinical data is limited [37••]. However, in general all studies report improvements in clinical outcomes for different meniscus implants. As meniscus replacements are not widely available and commonly used, the question arises whether these clinical improvements and the duration of these improvements are sufficient. Technical aspects (Table 2), such as fixation and difficulty of the surgical technique, might also affect market introduction and adoption. Replacing (part of) the lateral meniscus may be even more technically challenging than replacing the medial meniscus. Finally, business factors such as reimbursement and financial leeway are an important prerequisite for successful implementation.

**Table 2:** Technical characteristics of meniscus implants

Device	Partial / total	Type	Material	Fixation	
CMI	Partial	Degradable scaffold	Bovine collagen	Sutured to remaining meniscus rim	[34]
Actifit	Partial	Degradable scaffold	Polycaprolactone (80%) & polyurethane (20%)	Sutured to remaining meniscus rim	[84]
MeniscoFix	Partial	Degradable scaffold	p(DTD DD)^a^ fibers	Sutured to remaining meniscus rim	[48]
	Total	Degradable scaffold	p(DTD DD) fibers	Horn extension anchored in tunnel	[50]
NUsurface	Subtotal	Permanent implant	Polycarbonate polyurethane	No fixation	[53]
Artimis	Total	Permanent implant	Polycarbonate polyurethane	Fixation tape anchored in tunnel	[59]
Total Meniscus Replacement	Total	Permanent implant	Synthetic composite	Fixation tape anchored in tunnel	[60]

^a^Poly(desaminotyrosyl-tyrosine dodecyl ester dodecanoate)

### Working Mechanism

It is commonly known that tibiofemoral contact pressures significantly increase after meniscectomy [4, 61, 62] and that meniscal allograft transplantation leads to a reduction of these pressures [63, 64]. It is therefore no surprise that tibiofemoral contact mechanics have been evaluated for most of the meniscus implants mentioned in this review: Actifit, NUsurface, Artimis and MeniscoFix have all demonstrated to be able to decrease pressures after meniscectomy [50, 53, 59, 65]. If this biomechanical function is indeed the most important mechanism, the fact that even shrunk scaffolds or extruded allografts are sufficient to relieve pain, would mean that not much is needed to restore the balance of the knee joint. This theory may be supported by the fact that many post-meniscectomy patients do not require surgical intervention and can be successfully managed with pain medication [66]. For these patients, the biomechanical function is sufficiently maintained even after partial meniscectomy. The geometry of the tibial plateau and femoral condyle is found to be associated with knee degeneration [67]. In this study it is hypothesized that the increased cartilage stresses, caused by smaller contact surfaces, result in overloading of the joint. The final link from mechanical overload to pain is yet to be demonstrated but seems to be plausible.

Limited pre-clinical data is available on the effect of meniscus implants on other potential causes of post-meniscectomy pain. It is known that the menisci play a role in joint lubrication, partly by maintaining synovial fluid pressure, achieving very low friction coefficients [68]. One might hypothesize that removing part of the meniscus would reduce this fluid pressure, thus increasing the friction coefficient and with that the shear stresses on the cartilage and the subchondral bone, potentially leading to pain and cartilage wear. A partial or total meniscus implant could restore joint congruity and reduce friction, shear stresses and pain.

Another important function of the meniscus, knee joint stability [68], is partially lost after damage or removal of the meniscus, especially in the frequent concurrency with ACL deficiency [61]. Impaired knee stability may lead to increased stresses and strains on ligaments and other soft tissues, and to elevated shear stresses on the tibiofemoral cartilage [61]. A meniscus replacement might be able to relieve the resulting symptoms but, again, very limited evidence exists.

### Clinical Performance

Minimal clinically important differences (MCID) were established with respect to different PROMs after MAT [69]. Assuming that all other meniscus replacement techniques can be also deemed successful if these MCIDs are obtained, improvements in PROMs can be interpreted better. Recent studies indicate that Actifit and CMI achieve MCIDs in mean KOOS pain, Lysholm and IKDC at the latest follow-up, ranging from five to ten years [37••, 42, 47]. Improvements are already reported six months after implantation, and tend to stabilize after two years [36, 70]. NUsurface achieves MCIDs in mean KOOS pain score at six and 12 months, while long-term effectiveness remains to be demonstrated. MAT results in a MCID for KOOS pain and IKDC after five to 11 months for most patients [71], which is comparable to the timelines described for the different meniscus replacements. Therefore, it is unlikely that the clinical outcomes reported for meniscus replacements are deemed insufficient by the orthopaedic community and hold back clinical implementation.

### Survival & Fixation

A major prerequisite for success is survival of the implant; in case of scaffolds until sufficient ingrowth of tissue has occurred. The long-term survival required for the permanent meniscus replacements is yet to be demonstrated. The knee joint has proven to be a harsh environment, where the femur and tibia move in all directions and forces can reach up to five times body weight [72]. As it currently impossible to mimic the resected native meniscus in geometry and mechanical properties, local stresses caused by an imperfect fit can easily result in failure of an artificial meniscus.

Besides implant integrity, a main challenge is fixation of the device inside the knee joint. For the scaffolds, failure rates remain relatively high [37••], despite several studies to optimize suturing techniques and materials [73–75]. Due to the variability in failure criteria and availability of descriptive information, it is difficult to determine how often a malfunctioning fixation contributed to these failures [37••]. The rigidity of the fixation screws used with the Artimis meniscus prosthesis were identified as a major root cause for the high failure rate [58•, 59]. A much more flexible solution with suture tapes is intended to resolve the issue, but pre-clinical and clinical data is not available yet. NUsurface also started with a rigid fixation technique in an ovine animal study, i.e., stainless steel bolts at the anatomical location [76]. For reasons unknown to the authors, the anatomically shaped implant fixed with bolts was replaced by a discoid geometry with a high lateral edge for human applications [53]. The circumferential edge intends to keep the device in place, but did not prevent dislocation or rotation requiring surgical intervention for 11% of patients after one year [56•]. It becomes clear that a reliable fixation technique is vital for meniscus replacements, which may explain why the only published study on the novel Total Meniscus Replacement focuses on its dedicated fixation system [60].

### Surgical Procedure

All meniscus replacements described in this review can be inserted arthroscopically. Implantation of meniscal scaffolds is preceded by measuring the meniscal defect and cutting the device into shape [31, 75]. Fixation of the scaffold is an extensive procedure including different suturing techniques. The NUsurface free-floating meniscus implant does not require fixation, but the surgical technique is complicated by the use of trial implants in combination with intra-operative imaging [56•]. Moreover, the notchplasty that is frequently required significantly affects the extent and the difficulty of the surgical procedure [55]. The Artimis meniscus prosthesis does not require capsular fixation or notchplasty [59]. The surgery involves two bone tunnels for anchoring of the fixation tapes, strongly resembling ACL reconstruction techniques well-known to orthopaedic surgeons familiar with arthroscopic surgery. However, it is not yet known whether the current fixation technique is sufficient for long-term survival and how the surgical technique may be affected. All in all, the surgical procedures associated with implantation of meniscus replacements are not straightforward. This potentially contributes to slow adoption by the orthopaedic community, although MAT is also a challenging and lengthy procedure because of the many sutures required for capsular fixation [77, 78].

### Lateral Meniscus Replacement

Although medial meniscectomy is performed more often, higher rates of radiologic changes are found after lateral meniscectomy [79]. More MAT procedures are performed in the lateral compartment [80], indicating that there is also a patient need for artificial lateral meniscus replacements.

Remarkably, all companies developing total meniscus implants initially focus on replacing the medial meniscus. It is likely believed that it is easier to successfully replace the medial meniscus, due to the differences in anatomy and biomechanics of both menisci and knee compartments. The medial surface of the tibial plateau is concave, whereas the lateral side is convex, resulting in the so-called screw-home mechanism of the knee joint [81]. During flexion, this mechanism causes an anteroposterior displacement of the lateral meniscus of approximately 10mm, while the medial meniscus displaces less than half that distance [1]. If a meniscus replacement is fixated to the tibial plateau, larger displacements lead to higher strains and stresses in either the implant or its fixation. In all cases, more motion may lead in accelerated wear of the implant. Furthermore, the native anterior attachment site of the lateral meniscus merges with the ACL insertion, complicating anatomical fixation of a total meniscus implant.

While survival rates of medial and lateral meniscal allografts are similar, lateral MAT results in better clinical outcomes than medial MAT [80]. An extensive systematic review including a total of 568 patients found no differences between medial and lateral meniscal scaffolds in terms of clinical outcomes or survival rates after one to 14 years [82]. An even more recent study of the CMI implant analyzed 156 patients at a mean of 10 years of follow-up and did find that lateral implants had a higher risk (22.6%) of failing clinically or surgically [83]. In conclusion, lateral meniscus replacement might be more technically challenging, but also lead to better clinical outcomes, especially after total meniscectomy.

### Market Access and Adoption

Besides all technical challenges, there must be more reasons why meniscus implants are not generally available and commonly used yet. As indicated by Table 1, regulatory approval is not obtained easily and requires a long and expensive pathway. To gain traction in the market, commitment of leading orthopaedic surgeons and reimbursement by insurance companies are essential. For all these purposes, consistent and homogenous clinical data on a large group of patients is very important, but appears to be lacking [37••]. Furthermore, failure rates and other clinical outcomes may be improved by defining optimal inclusion criteria and concomitant interventions for these studies. However, the companies developing novel meniscus replacements are often start-up of spin-out companies, with very limited financial leeway. The resources of these small companies must be carefully allocated to develop a functional device, satisfy (potential) investors, and fulfill grant prerequisites. This leaves little time and budget for a strategy including multiple phases of clinical research, required to optimize the meniscus replacement, including the surgical technique and inclusion criteria. The validity of pre-clinical testing for validation purposes is limited, as it is not possible to accurately mimic the complex anatomy and kinematics of the large variety of knee joints that exists [67].

As all meniscus implants tend to improve clinical outcomes, it is important that future research does focus on determining the optimal treatment strategy for each patient population and consistently gathering clinical data on the effect of the interventions. Ideally, this would convince regulatory authorities, the orthopaedic community and insurance companies and prevent further shelving of promising meniscus replacement techniques.

## Conclusions

All meniscus replacements appear to have the potential to improve clinical outcomes of post-meniscectomy patients. However, homogenous data on the CMI and Actifit meniscal scaffolds is very limited, especially in the long-term. While the NUsurface implant is somewhat ahead of the Artimis prosthesis, long-term safety and efficacy remain to be demonstrated for both. It is of major importance that future research focuses on optimizing fixation methods and finding the optimal treatment strategy for each patient group. New techniques for partial and total replacement of the medial and lateral meniscus are rapidly developing and will be followed with interest.

## Data Availability

No datasets were generated or analysed during the current study.
